# Evaluation of Zoledronate for Pain Suppression in Diffuse Sclerosing Osteomyelitis of the Mandible: Protocol for a Prospective, Single-Arm Interventional Study

**DOI:** 10.7759/cureus.80739

**Published:** 2025-03-17

**Authors:** Junya Kusumoto, Shungo Furudoi, Daisuke Takeda, Megumi Kishimoto, Masaya Akashi

**Affiliations:** 1 Oral and Maxillofacial Surgery, Kobe University, Kobe, JPN; 2 Oral and Maxillofacial Surgery, Konan Medical Center, Kobe, JPN

**Keywords:** bisphosphonate, diffuse sclerosing osteomyelitis, pain suppression, prospective study, zoledronate

## Abstract

Introduction: Diffuse sclerosing osteomyelitis (DSO) is a non-bacterial osteomyelitis that occurs infrequently and is characterized by recurrent severe pain, swelling of the mandible, and trismus. To date, no effective treatment has been established. Bisphosphonates have been suggested as an efficacious treatment for DSO; however, no prospective studies have examined the effect of bisphosphonate treatment in adults with DSO. This study aims to evaluate the effect of zoledronate on pain suppression in patients with DSO.

Methods: This is a single-center, exploratory, single-arm, prospective interventional study. The participants will include eight patients with DSO of the mandible. The primary endpoint is the change in pain levels before and after zoledronate administration. The secondary endpoints are assessing the efficacy of the clinical course, imaging changes, and safety monitoring. Patients will be enrolled if they are deemed eligible following screening tests and will receive only one 4.0 mg intravenous dose of zoledronate within seven days of enrollment. Study participants will be followed up at one, four, 12, 24, and 48 weeks post-dose or at discontinuation after zoledronate administration.

Conclusion: This study is the first prospective trial to evaluate the effect of administering a single dose of zoledronate on pain suppression in patients with DSO. We believe this study will lead to the conclusion that zoledronate is an effective treatment for DSO.

## Introduction

Diffuse sclerosing osteomyelitis (DSO) is an infrequent non-bacterial disease of the bone. DSO of the mandible has an estimated prevalence of 1/200,000 [1] and is characterized by recurrent severe pain, swelling of the mandible, and difficulty opening the mouth [1,2]. Radiological examinations reveal diffuse sclerosis and partial osteolysis of the affected mandible [1,2]. DSO is also considered a type of synovitis, acne, pustulosis, hyperostosis, and osteitis (SAPHO) syndrome [3,4], which is a systemic disease proposed by Chamot et al. [5] in 1987; however, the etiology and pathophysiology of DSO remain unclear. Conventional treatment for DSO includes analgesics such as non-steroidal anti-inflammatory drugs (NSAIDs), antimicrobials, hyperbaric oxygen therapy, surgical treatment, and conservative treatment (occlusal sprint, physiotherapy, and myofeedback therapy) [6]. Clinically, symptomatic treatment with analgesics and antimicrobials is common; however, many cases relapse within a short period after showing improvement [7]. Long-term macrolide therapy is considered relatively effective; however, it has disadvantages. It takes time for the patient's condition to improve, complete remission is difficult to achieve, and patients are prone to relapse following drug discontinuation [2].

Intravenous bisphosphonates with bone resorption inhibitory activity are commonly used to inhibit bone resorption in bone metastases from solid tumors and improve hypercalcemia [8]. Bisphosphonates cause serious complications, such as medication-related osteonecrosis of the jaw (MRONJ), and risk factors such as malignancy, duration of antiresorptive therapy, use of corticosteroids, anemia, and diabetes mellitus have been cited [9]. Interestingly, in 2001, intravenous bisphosphonate (pamidronate) was reported to be effective in relieving bone pain in patients with DSO [10]. Since then, the efficacy of oral or intravenous bisphosphonates for treating DSO has been reported in several Japanese studies [11-17], and the development of MRONJ has not been reported. However, the use of oral or intravenous bisphosphonates as therapeutic agents for DSO is not currently approved, and the administration of bisphosphonates in such cases is considered off-label.

A systematic review has suggested that bisphosphonates are the most effective in improving DSO symptoms [6]. If conventional treatments do not improve the condition of patients with DSO, treating them with zoledronate, a bisphosphonate with strong bone resorption inhibitory properties, may be worthwhile. We obtained approval from the clinical ethics committee of Kobe University Hospital for the off-label use of zoledronate to treat DSO in November 2015 (certificate no. 67). Zoledronate was administered to six patients with DSO between November 2015 and February 2018. A single dose of zoledronate reduced mandibular pain in all six patients, and no serious adverse events, including MRONJ, were observed.

DSO causes severe and recurrent pain and a decline in quality of life. A treatment protocol has yet to be established for this disease; therefore, new, highly effective treatments that are fast-acting are required. To date, no prospective studies have examined the effects of bisphosphonate treatment in adults with DSO. Therefore, this study's main objective is to evaluate whether zoledronate has an effect on suppressing pain in patients with DSO who are aware of pain in the mandible.

This trial was retrospectively registered in the Japanese Trial Register (registration number jRCTs051180011).

## Materials and methods

Study design and setting

This exploratory, single-arm, prospective interventional study will only be conducted at Kobe University Hospital. The study will be conducted in a single-arm setting due to difficulties in securing a sufficient sample size owing to disease rarity and lack of standard treatment, as well as its status as a pilot trial for future research.

Patient selection

This study will include patients who meet all the following inclusion criteria. The inclusion criteria include patients aged 20-80 years at the time of consent to participate; diagnosed with DSO based on clinical symptoms, panoramic radiography, mandibular computed tomography (CT), magnetic resonance imaging (MRI), and blood test findings [2]; and who give their free and voluntary written consent to participate in this clinical study.

The exclusion criteria are patients without a diagnosis of DSO based on clinical symptoms, panoramic radiography, mandibular CT, MRI, and blood test findings and with a serum-corrected calcium (Ca) level of ≤8.6 mg/dL determined by a blood test before zoledronate administration. The exclusion criteria also include patients with hepatic dysfunction (serum aspartate aminotransferase (AST) or alanine aminotransferase (ALT) level ≥ 70 IU/L or total bilirubin (T-Bil) ≥ 2.0 mg/dL) and with renal dysfunction (estimated glomerular filtration rate (eGFR) < 30 mL/min). Patients with allergies or other drug sensitivities; a history of QT prolongation, seizures, tetany, numbness, and disorientation associated with hypocalcemia; and MRONJ or a history of MRONJ are also excluded from the study. The exclusion criteria also include patients with the following risk factors for MRONJ: malignancy, angiogenesis inhibitors, corticosteroid therapy, and radiation therapy; with symptoms of otitis externa, ear leakage, or ear pain; and on calcitonin preparations, aminoglycoside antimicrobials, or cinacalcet. Pregnant women and patients who may be pregnant or nursing, patients who participated in another clinical study within four months of participating in the current study, and patients deemed inappropriate for inclusion in the study by the investigators in charge of this clinical research are also excluded from the study.

Interventions

**Figure 1:**
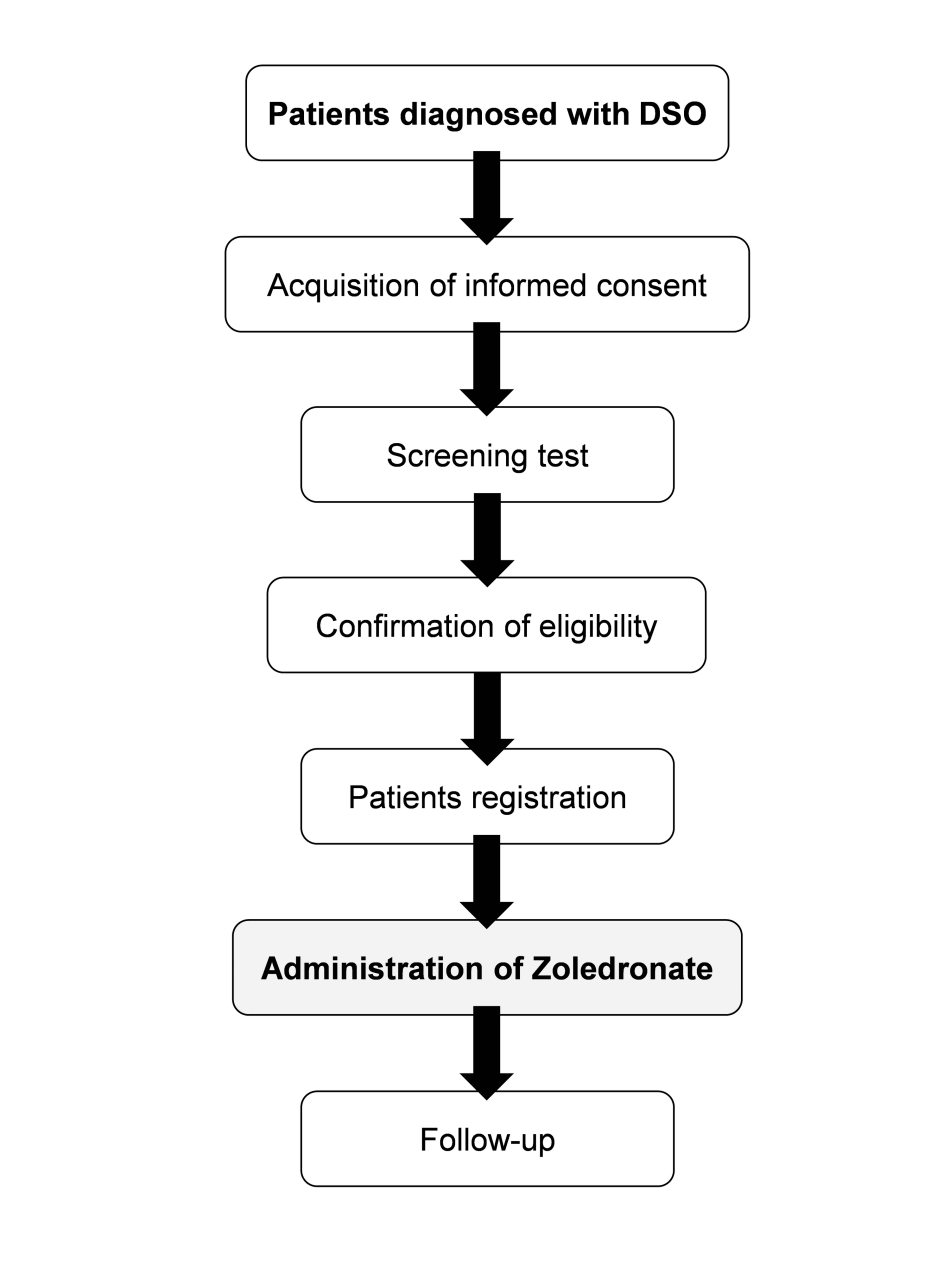
Flow chart of the study process to determine and manage the participants. DSO: diffuse sclerosing osteomyelitis

The study participants will be screened after their informed consent has been obtained. Screening tests include a patient background interview, recording of subjective and objective symptoms, vital signs (blood pressure, pulse, and temperature measurements), weight measurements, blood tests, and panoramic radiography and CT imaging. If necessary, analgesics such as NSAIDs and antibacterial agents will be used in combination therapy. Blood tests will be conducted to measure the white blood cell count, leukocyte fraction, red blood cell count, hemoglobin, hematocrit, platelet, AST, ALT, T-Bil, total protein, albumin, blood urea nitrogen (BUN), creatinine (Cr), eGFR, C-reactive protein, sodium, potassium, Ca, and magnesium. Patients will be enrolled if they are deemed eligible after screening tests have been completed and will receive only one 4.0 mg intravenous dose of zoledronate within seven days of enrollment (Figure 1). Zoledronate doses may be reduced according to the degree of renal function: 4.0, 3.5, and 3.0 mg for eGFR > 50, 40-50, and 30-49 mL/min, respectively.

The duration of the clinical study is approximately one year. The patients will be monitored one week before zoledronate administration; on the day of administration; at one, four, 12, 24, and 48 weeks after administration; and at the time of discontinuation. Prohibited concomitant medications include other bone resorption inhibitors (bisphosphonates and denosumab), and the concomitant use of these inhibitors is prohibited throughout this clinical study.

The clinical study will be terminated if continuation of the study is deemed impossible for any of the following reasons: First, the research participants request withdrawal from participation in the clinical study or withdraw their consent. Second, after enrolment, the participants do not satisfy the eligibility requirements. Third, continuing the clinical study due to worsening complications becomes difficult. Fourth, continuing the clinical study due to illness, etc., becomes difficult. Fifth, the entire clinical study is terminated.

Endpoints

The primary endpoint is the amount of change in the mandibular pain assessment index (Visual Analog Scale (VAS): 0-10) from baseline to 48 weeks after treatment (0: no pain; 10: no more pain experienced) [18]. The secondary endpoints are efficacy endpoints including the duration of the pain suppression effect, the extent to which the participant can open their mouth, and the presence or absence of bone with a worm-eaten appearance (indicating resorption) on CT imaging and safety endpoints including the results of blood test results (BUN, Cr, eGFR, and Ca levels) and side effects of zoledronate.

Patient clinical management and follow-up

Study participants will be followed up at one, four, 12, 24, and 48 weeks post-dose or at discontinuation. Self-reported and other symptoms, vital signs, weight measurements, and blood tests will be recorded at each visit. In addition, panoramic radiography or CT imaging will be performed 24 and 48 weeks after administration. Concomitant therapy with analgesics, including NSAIDs and antimicrobials, will be administered, when necessary, for the duration of the clinical study. The different time points are listed in Table 1.

**Table 1:** Implementation schedule and procedures ○: items to be performed before zoledronate administration; ■: zoledronate administration; ​​​​​​​●: items to be performed following zoledronate administration; ​​​​​​​*: concomitant use of analgesics, including non-steroidal anti-inflammatory drugs and/or antimicrobials

	Screening test	Date of administration	Observation period				
Time point	Before 1 week	0	After 1 week	After 4 weeks	After 12 weeks	After 24 weeks	After 48 weeks
Consent acquisition	○						
Check patient background	○						
Zoledronate administration		■					
Check of subjective and objective symptoms	○	●	●	●	●	●	●
Observation of side effects	○	●	●	●	●	●	●
Vital sign measurement	○	●	●	●	●	●	●
Weight measurement	○	●	●	●	●	●	●
Blood tests conducted	○		●	●	●	●	●
Panoramic radiography conducted	○					●	●
Computed tomography conducted	○					●	●
Concomitant therapy administered^*^	○	●	●	●	●	●	●

Sample size

The target number of participants is eight. Among the reasons for this setting is that the frequency of DSO is extremely low, and only six patients have been administered zoledronate in the past two years in our department. In addition, this clinical study has an exploratory aspect; therefore, the target number of eight cases is considered reasonable. According to the report by Otto et al. [18], a rationale for setting this parameter is that if the changes in the means and standard deviations of the VAS are considered to be six, a one-sample t-test with a significance level of 5% (two-tailed) would yield a power of 80% in eight cases.

Data collection and management

In this clinical study, the clinical research database and clinical research-related documents will be associated with the original data of the research participants using a list of research participant identification numbers. The principal investigator or sub-investigator will prepare a case report form (CRF) for each research participant. The CRF will include the date on which participant consent was obtained, participant identification number, sex, date of birth, age at consent, date of observation, subjective and objective symptoms, vital signs, weight, imaging findings, and blood test results.

Important documents related to the conduct of the research (e.g., materials or records supporting the information used in the study) will be stored in a lockable place for 10 years after the research is discontinued or terminated or 10 years from the date of publication of the research results, such as papers, whichever is later. Thereafter, the documentation will be disposed of in a manner that does not allow the identification of individuals.

Data monitoring

Monitoring will be conducted semi-annually to verify that this clinical research is being conducted safely and in accordance with the protocol and that data is being accurately accumulated. A dedicated staff member will supervise this work.

Statistical analysis

The analysis will be performed after the study drug administration has been completed in all cases and the data have been fixed. For the main analysis, a one-sample t-test will be used to calculate p-values for the change in the pain assessment index (VAS) from one week before to 48 weeks after treatment administration. The significance level is assumed at 5%. For the secondary analysis, the median duration of pain relief, evaluation of the extent to which participants can open their mouths, the percentage of bone resorption measured using CT imaging, and the median value of the duration of the pain reduction effect, as well as their 95% confidence intervals, will be calculated. In addition, changes in the pain assessment index (VAS), BUN, serum Cr, eGFR, and serum Ca levels over time at each time point will be graphed.

We anticipate that missing data are unlikely to arise from the endpoints of the study. However, if significant missing data arise that may have affected the results, they will either be excluded from the study or treated with multiple imputations assuming missing at random.

Ethics approval and consent to participate

Currently, bisphosphonates are used off-label in DSO. Zoledronate is currently being used as an investigational drug for DSO as part of a physician-guided clinical trial in our department. This study was independently reviewed and approved by the Kobe University Clinical Research Ethics Committee (certificate no. C180005) and is being conducted in accordance with the Declaration of Helsinki. Written informed consent will be obtained from all the participants.

## Results

The study began in April 2018 and will continue until March 2026. Patients and the public are not directly involved in establishing the research question, study design, outcome measures, recruitment, or conduct of the study. Tables will be prepared for the endpoints; the exact 95% confidence intervals of the binomial distribution will be calculated for each group to compute percentage estimates.

It is hypothesized that the osteolysis of the DSO imaging findings would be associated with symptoms such as pain. It is expected that zoledronate administration, a potent bone resorption inhibitor, will reduce pain by decreasing the osteolysis area. The amount of change in pain before and after the administration of zoledronate is assessed; the closer the VAS value approaches 0 after zoledronate administration, the stronger the pain-suppressing effect of zoledronate on DSO is explained. The change in pain over time is expressed graphically. Moreover, it is expected that zoledronate has an immediate effect on DSO if the pain suppression effect develops early after administration. Concomitantly, if CT images after zoledronate administration show a decrease in the osteolysis area, the decrease in osteolysis is considered to be due to zoledronate administration. In addition, zoledronate is an existing medicine, which explains the safety of zoledronate in patients with DSO if zoledronate administration does not cause serious side effects.

The results of this study will be published in a peer-reviewed international journal. We also plan to present at a scientific meeting to share the results with other researchers in Japan.

## Discussion

The main objective of this study is to determine the efficacy of administering a single dose of zoledronate to suppress pain in patients with DSO. To the best of our knowledge, no prospective studies have suggested the efficacy of zoledronate in the treatment of DSO; thus, this is the first trial.

According to the recent systematic review [6], NSAIDs provide early symptomatic improvement in DSO of the mandible; however, the complete remission rate for anti-inflammatory drugs including steroids is 2%. In addition, the complete remission rate is 19% for antimicrobials, including long-term macrolide therapy. On the other hand, bone resorption inhibitors are more effective for DSO of the mandible (the complete remission rate, 45%). Most of the bone resorption inhibitors reported to be effective in DSO treatment are bisphosphonates, particularly pamidronate, a non-cyclic-type nitrogen-containing bisphosphonate [7,10,11,13,14,19,20]. All reports of pamidronate administration for treating DSO were case reports or retrospective case-control studies, and the doses and number of administrations differed. In many cases, the effect was achieved within a few days of the start of the administration of the agent, and multiple doses were administered. Zoledronate, a cyclic-type nitrogen-containing bisphosphonate, has been reported to inhibit bone resorption more than 300 times more potently than pamidronate [21]. Therefore, we hypothesized that only one dose of zoledronate might be effective for treating DSO. In a preliminary study, we administered zoledronate to six patients with DSO. We found that pain resolved within 24 hours after administration in most patients, with no symptom relapse observed after only a single dose. Thus, we designed the current study.

MRONJ is a serious side effect of bisphosphonates [9]. Bisphosphonate-induced MRONJ is likely to occur with long-term intravenous administration [9,22], but to our knowledge, MRONJ has not been reported to occur after only one dose of zoledronate. Therefore, administering a single dose of zoledronate to treat DSO could show benefits in terms of the occurrence of side effects, even though MRONJ development would still be of concern.

The radiographic findings of DSO show an osteolytic area that is considered to be responsible for causing clinical symptoms such as pain and swelling [1]. Since a histological study of patients with acute-phase DSO has suggested that many osteoclasts develop and produce cathepsin K [23], the osteolytic area is probably a site of over-activated osteoclasts. The mechanism through which DSO develops is not yet clear; however, suppressing activated osteoclasts may lead to symptom alleviation. Since bone resorption inhibitors such as bisphosphonates inhibit osteoclasts, they may exert a pain suppression effect in patients with DSO by affecting the osteolytic area. No treatment regimen for DSO has been established; however, we believe this study will lead to zoledronate being an effective treatment for DSO.

The main limitation of this study protocol is the lack of a set control group. Although a comparison with a placebo control would be more beneficial, the rarity of the disease makes recruiting a sufficient number of patients difficult. Therefore, although the study will be conducted as a single-arm study, the potential placebo effect cannot be completely ruled out. Alternatively, comparison with historical controls may be useful; however, it will not be considered in this study as there is currently no established treatment for DSO other than symptomatic treatment. Future research efforts will build on the results found in this study and aim to accumulate more cases and expand the scope through multicenter studies.

## Conclusions

This study is the first prospective trial to evaluate the effect of administering a single dose of zoledronate on pain suppression in patients with DSO. Since acute symptoms of DSO are considered to be associated with areas of osteolysis, and zoledronate is a potent bone resorption inhibitor, we hypothesized that zoledronate would be a highly effective agent to treat DSO. We believe this study will lead to zoledronate being an effective treatment for DSO.
